# Long-term exposure to low-dose *Haemophilus influenzae* during allergic airway disease drives a steroid-resistant neutrophilic inflammation and promotes airway remodeling

**DOI:** 10.18632/oncotarget.24653

**Published:** 2018-05-18

**Authors:** Xu Yang, Yijie Wang, Shengtao Zhao, Ran Wang, Changzheng Wang

**Affiliations:** ^1^ Institute of Respiratory Disease, Xinqiao Hospital, Third Military Medical University (Army Medical University), Chongqing, 400037, China; ^2^ Department of Respiratory Medicine, The 305 Hospital of PLA, Beijing, 100017, China; ^3^ Department of Respiratory Medicine, Kunming General Hospital of Chengdu Military Region, Kunming, 650032, China

**Keywords:** allergic airway disease, Haemophilus influenzae, neutrophilic inflammation, airway remodeling, long-term effect, Immunology

## Abstract

Growing evidences indicate that bacteria are associated with pathogenesis of neutrophilic asthma. However, the long-term effect of airway bacterial colonization remains unclear. We sought to establish a murine model to simulate the airway inflammation of long-term bacterial colonization, and to assess the effects of bacteria on allergic airway disease (AAD). BALB/c mice were sensitized twice and subsequently challenged with ovalbumin (OVA) and exposed to low-dose *Haemophilus influenzae* for approximately 2 months. Mice in treatment groups inhaled budesonide for consecutively 6 days in the last week. Airway inflammatory phenotype, immune response, phagocytic capacity, mucus production, airway remodeling and steroid sensitivity were assessed. Long-term exposure to low-dose *H. influenzae* during AAD did not cause serious infection but only a slightly increased airway inflammation, which resembled the colonization. Inflammatory phenotype was converted from a steroid-sensitive T helper (Th) 2-associated eosinophilic inflammation to a steroid-resistant Th17-associated neutrophilic inflammation. The increased neutrophilic inflammation was accompanied by defects in regulatory T cell (Treg)-associated immunosuppression and macrophage phagocytosis, and finally promoted mucus hypersecretion and airway remodeling. These features resembled those of refractory neutrophilic asthma in humans. These findings indicate that in asthmatic patients, airway bacterial colonization may be a potential therapeutic target. Minimizing the pathogen burden in airway, such as *Haemophilus influenzae*, may be beneficial.

## INTRODUCTION

Asthma is now recognized as a chronic heterogeneous airway inflammatory disease. Although Th2-derived eosinophilic inflammation is the hallmark of asthma, a typical eosinophilic inflammation is found absent in nearly 50% of asthmatics [1]. Neutrophilic asthma is a subgroup of non-eosinophilic asthma [2], and is characterized by an intense increase in airway neutrophils, severe disease conditions [3], airflow limitations [4], and poor response to steroid treatments [5]. These severe and treatment-resistant cases of asthma bring a heavy financial burden, thus better understanding of the pathogenesis and novel therapies are needed.

Recent years, with the development of culture-independent molecular technology, the lower airway is no longer thought sterile [6]. Although asthma is usually known as a non-infectious allergic disease, a growing number of evidences have linked it to microbes. Compared with healthy controls, patients with asthma have greater airway bacterial burdens [7]. And the burdens present even higher in asthmatics with lower levels of Th2-related airway inflammation [8]. Furthermore, the airway bacterial community composition, which has been demonstrated different between healthy controls and asthmatic patients [9], varies with disease features, steroid responses and inflammatory phenotypes of asthma [8, 10, 11]. About 60% of patients with neutrophilic asthma have pathogenic microorganisms cultured from their bronchoalveolar lavage fluids (BALF)[12]. Detection of bacteria in the lower airways is associated with acute wheezy episodes [13], enhanced neutrophilic airway inflammation [14], and impaired pulmonary function [15], which can be improved by antibiotics treatment [16]. These findings suggest that bacterial colonization in the airway in asthma is much more common than it was expected and probably plays a role in pathogenesis of neutrophilic asthma.

The effects of bacteria can be dose and species dependent [17, 18]. Although transient bacterial infection has been reported to be able to induce a steroid-resistant chronic neutrophilic inflammation in a period of time [19], whether and how a long-term colonization of bacteria with a low dose in the airway would affect the progression of asthma is yet unknown.

Unlike infection, airway bacterial colonization is often subclinical and free of exaggerated symptoms. And bacterial burdens in the airway can be variable over time, even in a same person. The two factors both make it difficult to identify a group of patients who have long-term colonization by enough loads of bacteria, thus characteristics of these patients is hard to be determined. On the other hand, ethical issues won't allow direct administration of certain bacterial species to humans to investigate their influences on AAD on mechanism level. Therefore, studies of this kind must be done using animal models.

The aim of the present study was to establish a murine model to simulate the slight inflammation of long-term airway bacterial colonization, and investigate the long-term effects of bacterial exposure on OVA-induced AAD, to elucidate the potential association between bacterial colonization and features of neutrophilic asthma. *Haemophilus influenzae*, the predominant pathogen involved in neutrophilic asthma [20] and associated with significantly higher levels of airway inflammation than other common pathogens [21], was applied in the study.

## RESULTS

### Transient exposure to *H. influenzae*

In the present study, we first sought to determine the effects of transient exposure to *H. influenzae* and find an appropriate dose for *H. influenzae*, to establish a further long-term model without serious infection. For this, non-allergic mice were intranasally inoculated with a series doses of *H. influenzae*, from 1 × 10^5^ to 1 × 10^8^ colony-forming units (CFU).

Whatever the dose was, all mice inoculated were survived. However, symptoms were different among groups. Mice exposed to 10^8^ CFU showed a serious piloerection and rigor, and were in a hunched posture. Those exposed to 10^7^ CFU showed a mild piloerection and a lack of grooming. In comparison, mice in 10^6^ and 10^5^ CFU groups had no obvious symptoms.

**Figure 1:**
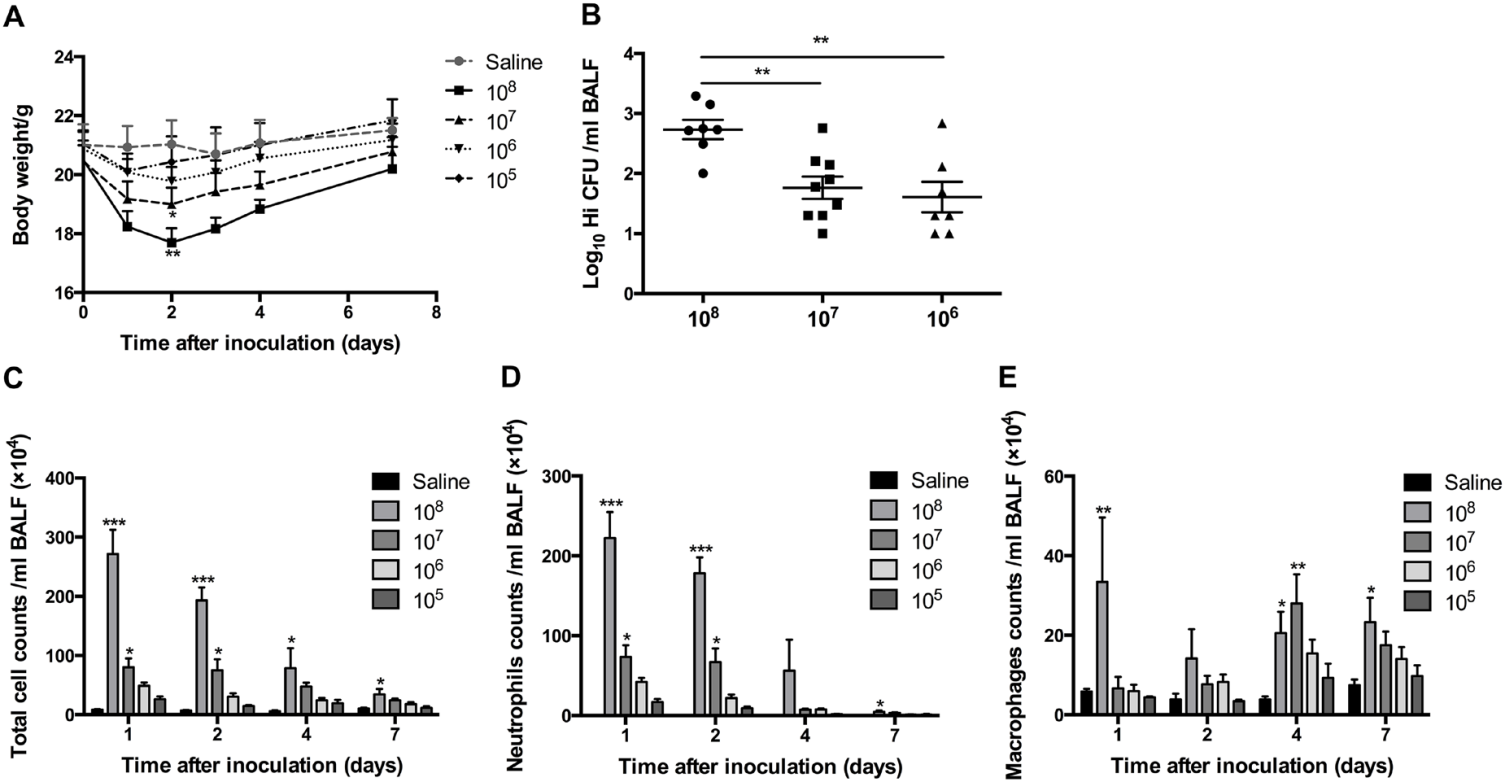
The effects of transient exposure to different doses of *H. influenzae* Mice were inoculated intranasally with 1 × 10^5^ to 1 × 10^8^ CFU of *H. influenzae* or saline. The effects of different doses of *H. influenzae* on body weight changes **(A)**, bacterial recovery from the BALF at day 1 postinfection **(B)**, and numbers of total cell **(C)**, neutrophil **(D)** and macrophage **(E)** in the BALF at days 1, 2, 4 and 7 postinfection were assessed. Data presented the mean ± SEM. Significant differences between saline and inoculation groups or within the inoculation groups are shown as ^***^P<0.001, ^**^P<0.01, ^*^P<0.05. Hi: *Haemophilus influenzae*.

There was a direct correlation between bacterial dose and weight loss. Body weight dropped to the lowest level at day 2 postinfection. As expected, mice in 10^8^ CFU group had the most significant weight loss, but by lowering the bacterial CFU constant, the weight loss decreased. At day 7 postinfection, weights of almost all mice returned to the level of baseline (Figure 1A).

There were no detectable viable *H. influenzae* in the BALF of mice receiving the dose of 10^5^ CFU, even if at the first day postinfection. In comparison, more than 70% of mice in another three groups recovered *H. influenzae* from BALF at day 1 postinfection. Mice in 10^8^ CFU group recovered much greater numbers of *H. influenzae* than those in 10^7^ and 10^6^ CFU group (Figure 1B). However, at days 2, 4 and 7 postinfection, whatever the dose of *H. influenzae* inoculated was, the proportions of mice with bacterial viable detection were less than 20%. The highest proportion, which was 40% and correlated with a mean recovery number of 10^1.15^ CFU/ml in the BALF, occurred at day 7 postinfection in 1 × 10^6^ CFU group.

*H. influenzae* of 10^8^ and 10^7^ CFU led a transient significant increases in BALF cell numbers. Total cell and neutrophil numbers peaked at day 1 postinfection, then declined to the levels around baseline at day 7 (Figure 1C, 1D); while macrophage number increased progressively and became higher at days 4 and 7 postinfection (Figure 1E). Mice in 10^6^ and 10^5^ CFU groups showed the same trends but not significant (Figure 1C-1E).

Following above observations, 1 × 10^6^ CFU, which had viable recovery of *H. influenzae* but no obvious symptoms, weight loss and inflammatory cell increase, was chosen as the dose for further application.

### Long-term exposure to low-dose *H. influenzae* combined with or without allergic airway disease does not cause serious infection

**Figure 2:**
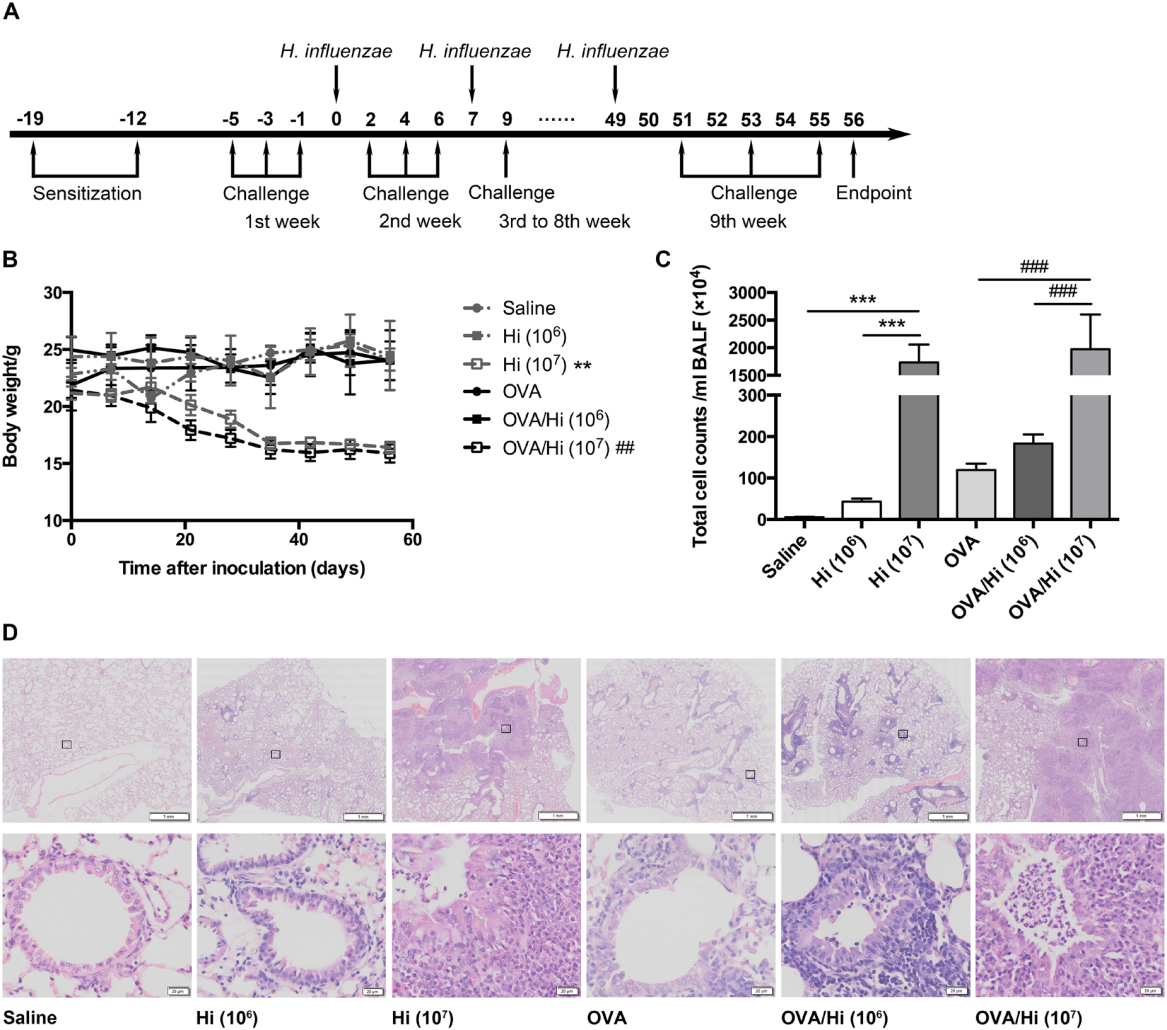
Long-term exposure to low-dose *H. influenzae* combined with or without allergic airway disease does not cause serious infection Mice were sensitized twice (days -19 and -12) and challenged three times a week (day -5 to 55) with OVA to establish and maintain an allergic airway inflammation. Respectively, 1 × 10^6^ (selected) and 1 × 10^7^ (positive control) CFU of *H. influenzae* were administered 24 hours after the third challenge every week (day 0 to 49). Seven days after the 8th administration and 1 day after the last challenge (day 56), mice were sacrificed **(A)**. To estimate the severity of infection, changes in body weights **(B)**, total cell counts in the BALF **(C)**, and lung histopathology (**D**, original magnification: upper, ×8; lower, ×200) were assessed. Data presented the mean ± SEM. Significant differences between groups without AAD are shown as ^***^P<0.001, ^**^P<0.01. Significant differences between groups with AAD are shown as ^###^P<0.001, ^##^P<0.01. For body weights change evaluation, P value was calculated by one-way repeated measures ANOVA and was for the entire time-related weight. Hi: *Haemophilus influenzae*.

To investigate the effects of long-term exposure to *H. influenzae* on AAD, mice were intranasally inoculated with the selected dose of *H. influenzae* (1 × 10^6^ CFU) 24 hours after the last OVA challenge every week. The effects were assessed after 8-week inoculation and 9-week challenges (Figure 2A). To better estimate the inflammatory response of long-term exposure to the selected dose of *H. influenzae*, we also set positive controls, in which mice were exposed to 1 × 10^7^ CFU of *H. influenzae*.

During the time from the first inoculation with 1 × 10^6^ CFU of *H. influenzae* to the endpoint, allergic and non-allergic mice (OVA/Hi and Hi groups) both showed no significant changes in their body weights compared with mice that were not inoculated with bacteria (OVA and saline groups). In comparison, the 8-week exposure to 1 × 10^7^ CFU of *H. influenzae* resulted in significantly progressive weight decreases (Figure 2B).

Both allergic and non-allergic mice exposed to 1 × 10^7^ CFU had more than 1500 × 10^4^ cells/ml of BALF, which were much higher than those exposed to 0 or 1 × 10^6^ CFU, which had lower than 300 × 10^4^ cells/ml of BALF (Figure 2C).

The histopathology of lungs showed similar trends. Mice in saline group showed no pathology. Mice with AAD (OVA group) had some inflammatory cells, including eosinophils, around the bronchioles. Compared with the corresponding controls, mice with long-term exposure to 1 × 10^6^ CFU of *H. influenzae* had a mild increased inflammation. However, infiltration of inflammatory cells was significantly aggravated by long-term exposure to 1 × 10^7^ CFU of *H. influenzae* (both Hi and OVA/Hi groups) with apparent alveolar consolidation occurred (Figure 2D).

In addition, mice with long-term inoculation with 1 × 10^6^ CFU of *H. influenzae* had no symptoms. But 28.3% of mice treated with 1 × 10^7^ CFU died from serious infection before endpoint.

These results suggested that long-term exposure to low-dose (1 × 10^6^ CFU) *H. influenzae* did not cause serious infections in both allergic and non-allergic mice, and successfully simulated the slight airway inflammation of bacterial colonization.

### Long-term exposure to low-dose *H. influenzae* during allergic airway disease converts a steroid-sensitive Th2-associated eosinophilic inflammation to a steroid-resistant Th17-associated neutrophilic inflammation

**Figure 3:**
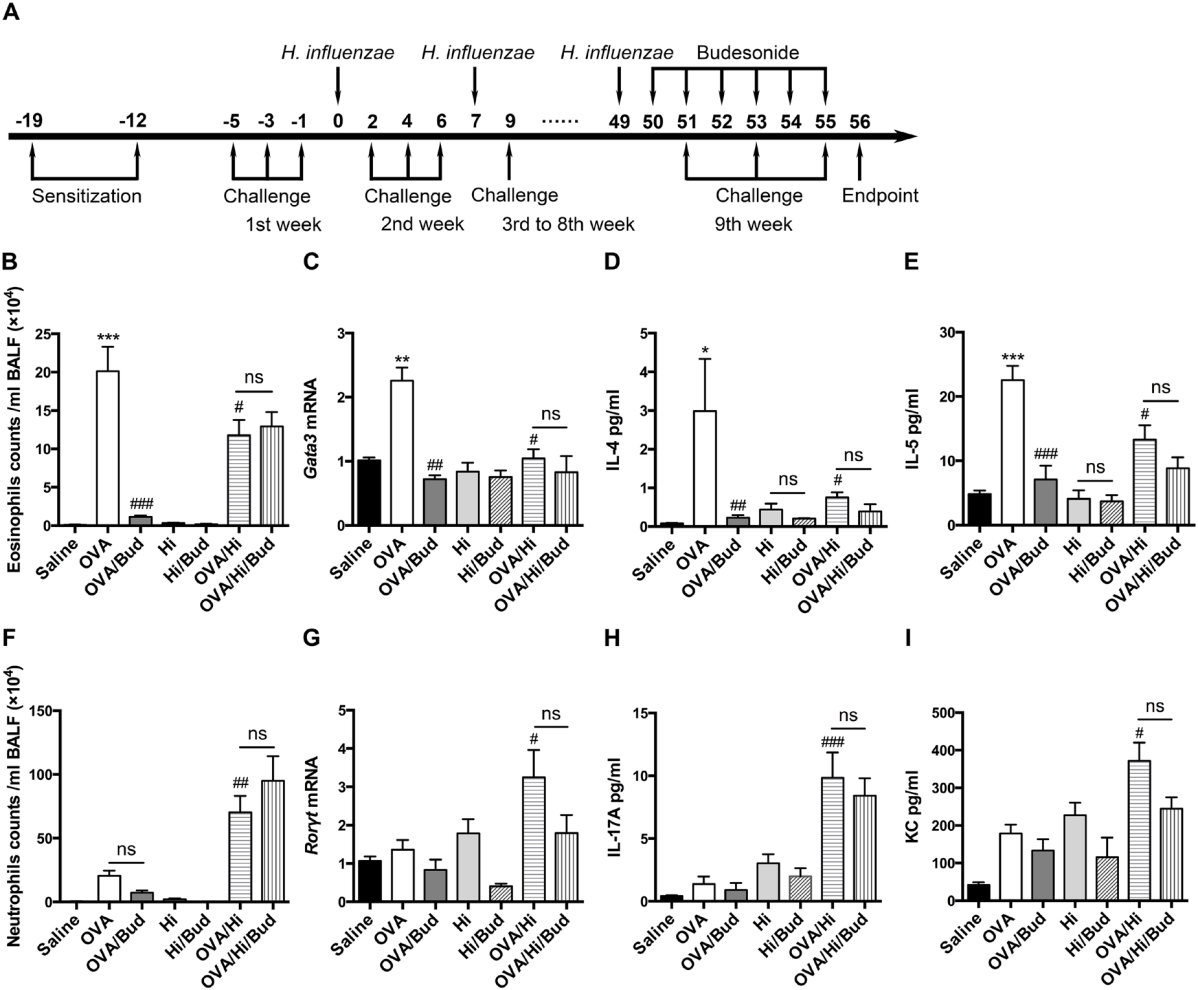
Long-term exposure to low-dose *H. influenzae* during allergic airway disease converts a steroid-sensitive Th2-associated eosinophilic inflammation to a steroid-resistant Th17-associated neutrophilic inflammation AAD was established and maintained by OVA sensitization twice and challenge three times a week. Long-term exposure to low-dose *H. influenzae* was administered by intranasally inoculation with 1 × 10^6^ CFU of *H. influenzae* once a week. Budesonide was given via inhalation once per day for 6 consecutive days just before endpoint **(A)**. Eosinophil number **(B)** in BALF, *Gata3* mRNA expression **(C)** in lung tissue, and levels of IL-4 **(D)** and IL-5 **(E)** in BALF were assessed to evaluate Th2-associated eosinophilic inflammation. Neutrophil number **(F)** in BALF, *Rorγt* mRNA expression **(G)** in lung tissue, and levels of IL-17 **(H)** and KC **(I)** in BALF were assessed to evaluate Th17-associated neutrophilic inflammation. Data presented the mean ± SEM. Significant differences between saline group and the others are shown as ^***^P<0.001, ^**^P<0.01, ^*^P<0.05. Significant differences between OVA groups and the others are shown as ^###^P<0.001, ^##^P<0.01, ^#^P<0.05. No significance was ns for short. Hi: *Haemophilus influenzae*; Bud: budesonide.

As described above, AAD was established and maintained by OVA sensitization twice and challenge three times a week. *H. influenzae* was administered by intranasally inoculation with 1 × 10^6^ CFU of *H. influenzae* once a week. To investigate the effects of steroid treatment, budesonide was given via inhalation once per day for 6 consecutive days just before endpoint. (Figure 3A).

Long-term exposure to low-dose *H. influenzae* significantly modified the airway inflammatory phenotype in mice with AAD. The induction of AAD (OVA group) resulted in airway eosinophilic inflammation, accompanied by an elevation in *Gata3* (Th2 differentiation factor) mRNA expression in lung tissue and increases in interleukin (IL)-4 and IL-5 (Th2-associated cytokines) levels in BALF compared with saline controls (Figure 3B-3E). However, long-term exposure to *H. influenzae* during AAD (OVA/Hi group) significantly reduced the eosinophil number in BALF (Figure 3B). This reduction was accompanied by down-regulation in *Gata3* mRNA expression in lung tissue (Figure 3C). Levels of IL-4 and IL-5, which were involved in eosinophil recruitment, were also significantly decreased in BALF of mice with AAD combined bacterial exposure compared with AAD alone (Figure 3D-3E).

In the meanwhile, the mild airway neutrophilic inflammation in AAD was significantly enhanced and became predominant when combined with long-term exposure to *H. influenzae*. Numbers of neutrophils in BALF were significantly increased in OVA/Hi group vs. OVA group and this increase was not shown in non-allergic mice exposed to *H. influenzae* alone (Figure 3F). In addition, compared with AAD alone, expression of Th17 differentiation factor, *Rorγt*, was significantly up regulated by bacterial exposure during AAD in lung tissue (Figure 3G). Mice exposed to *H. influenzae* during AAD also had an elevated level of IL-17 in BALF than those were not exposed (Figure 3H). Keratinocyte chemokine (KC), which is the mouse ortholog of human IL-8 and can be induced by IL-17 to recruit neutrophils, also showed significant increase in the BALF of mice in OVA/Hi group compared with OVA group (Figure 3I).

After budesonide treatment, Th2-associated eosinophilic inflammation in mice with AAD alone (OVA/Bud group) was significant suppressed. In contrast, budesonide treatment did not cause reductions of all above key features of allergic airway inflammation in mice combined AAD with long-term exposure to *H. influenzae* (OVA/Hi/Bud group) (Figure 3B-3E).

Collectively, our results showed that long-term exposure to *H. influenzae* during AAD might convert the airway inflammation from a steroid-sensitive Th2 associated eosinophilic phenotype to a steroid-resistant Th17 associated neutrophilic phenotype, which resembles the features of neutrophilic asthma in human.

### Long-term exposure to low-dose *H. influenzae* during allergic airway disease inhibits Treg-associated immunosuppression but does not affect Th1-associated inflammation

**Figure 4:**
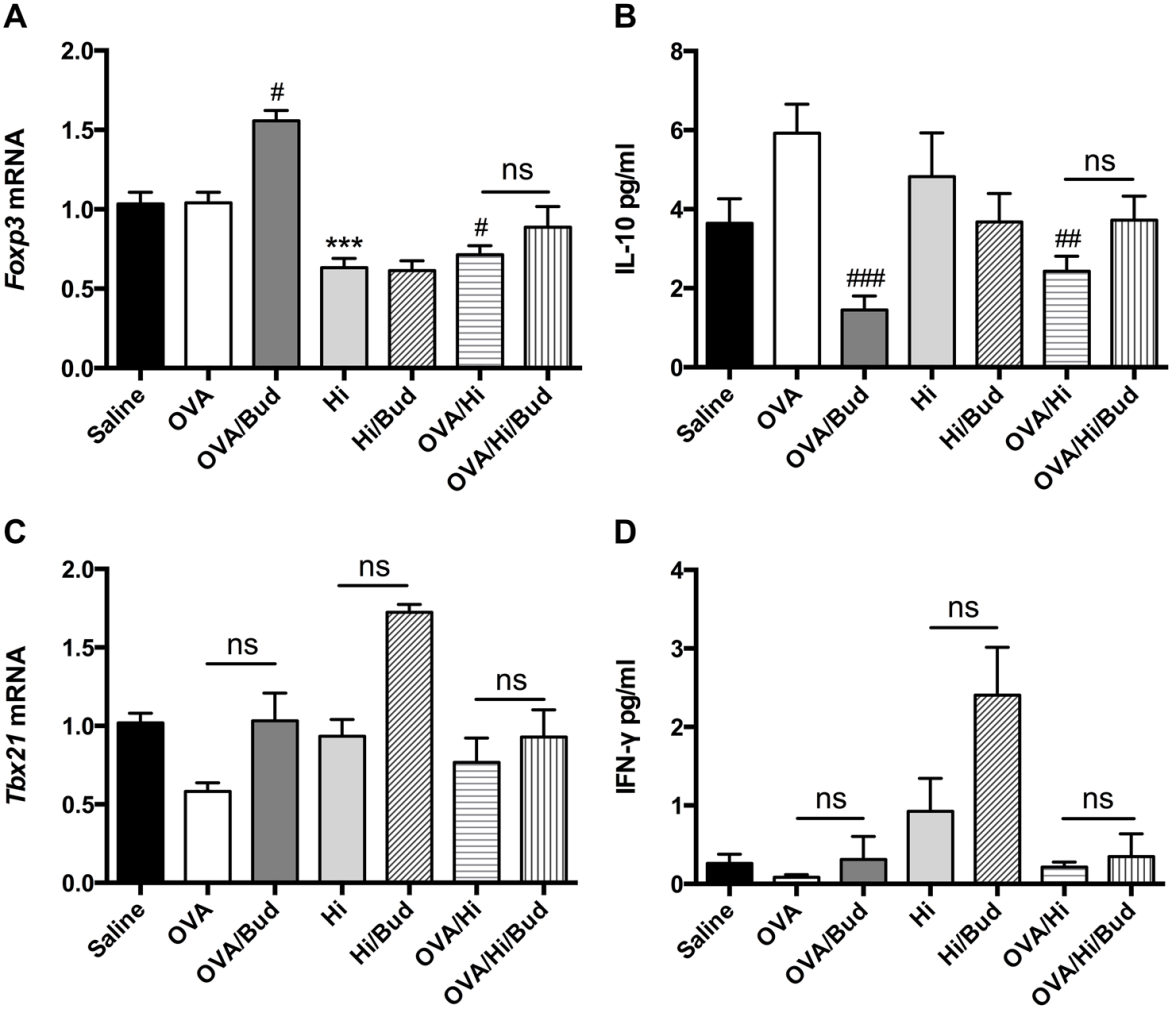
Long-term exposure to low-dose *H. influenzae* during allergic airway disease inhibits Treg-associated immunosuppression but does not affect Th1-associated inflammation To determine the role of Treg-associated immunosuppression and Th1-associated inflammation, expressions of *Foxp3* **(A)** and *Tbx21* **(C)** mRNAs in lung tissue, levels of IL-10 **(B)** and IFN-γ **(D)** in BALF were assayed. Data presented the mean ± SEM. Significant differences between saline group and the others are shown as ^***^P<0.001. Significant differences between OVA groups and the others are shown as ^###^P<0.001, ^##^P<0.01, ^#^P<0.05. No significance was ns for short. Hi: *Haemophilus influenzae*; Bud: budesonide.

To determine whether long-term exposure to *H. influenzae* during AAD had effects on Tregs, expression of *Foxp3*, the Treg differentiation factor, and level of IL-10, a key anti-inflammatory cytokine mainly produced by Tregs, were assayed. Compared with AAD alone, bacterial exposure during AAD significantly down regulated the expression of *Foxp3* mRNA in lung tissue (Figure 4A). Consistent with that, IL-10 level in BALF was decreased in OVA/Hi group vs. OVA group (Figure 4B). This indicated that long-term bacterial exposure during AAD might cause a defect in anti-inflammatory response. Although treatment with budesonide significantly enhanced the expression of lung *Foxp3* mRNA in mice with AAD alone (OVA/Bud group), mice with AAD combined bacterial exposure (OVA/Hi/Bud group) revealed poor response to the treatment (Figure 4A). Similarly, IL-10 level in BALF showed significant decrease after budesonide treatment in mice with AAD alone because of the strong anti-inflammatory effect of steroids, while it was found no decrease after budesonide treatment in allergic mice with long-term bacterial exposure (Figure 4B).

To determine the effects of long-term exposure to *H. influenzae* during AAD on Th1-associated inflammation, expression of *Tbx21* mRNA and level of interferon (IFN)-γ were assessed. They both had trends of down-regulations in OVA group vs. saline group and up-regulations in OVA/Hi group vs. OVA group. However, these changes were not significant (Figure 4C-4D). Treatments with budesonide tended to increase their expressions in mice in OVA/Bud, Hi/Bud and OVA/Hi/Bud groups compared with their corresponding controls, but no significant differences were found either (Figure 4C-4D).

Collectively, these results indicated that there existed an imbalance between pro-inflammatory response and anti-inflammatory response after long-term exposure to *H. influenzae* during AAD, which might be involved in steroid resistance. However, no significant effects on Th1-associated inflammation were found.

### Long-term exposure to low-dose *H. influenzae* during allergic airway disease impairs the phagocytosis by macrophage

**Figure 5:**
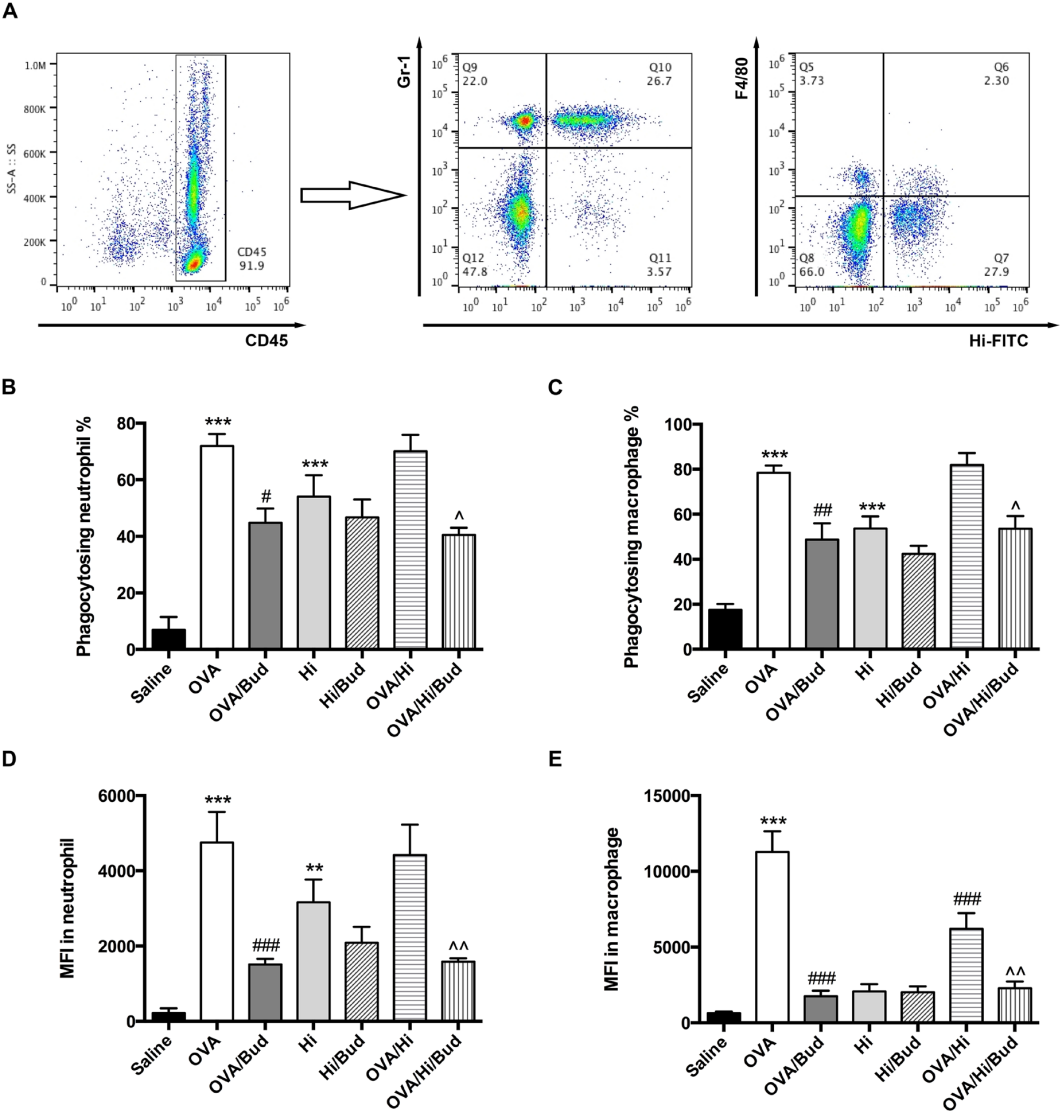
Long-term exposure to low-dose *H. influenzae* during allergic airway disease impairs the phagocytosis by macrophage BALF cells were obtained. Phagocytic capacities of *H. influenzae* by both neutrophil and alveolar macrophage in BALF were assessed using flow cytometry. Representative scatter plots were shown **(A)**. Among the CD45+ cells, neutrophil was further identified as high expression of Gr-1 while macrophage was further identified as high expression of F4/80. The effects on proportion of phagocytosing neutrophil **(B)**, proportion of phagocytosing macrophage **(C)**, MFI of FITC in neutrophil **(D)** and MFI of FITC in macrophage **(E)** were assessed. Data presented the mean ± SEM. Significant differences between saline group and the others are shown as ^***^P<0.001, ^**^P<0.01. Significant differences between OVA groups and the others are shown as ^###^P<0.001, ^##^P<0.01, ^#^P<0.05. Significant differences between OVA/Hi and the others are shown as ^^P<0.01, ^P<0.05. Hi: *Haemophilus influenzae*; Bud: budesonide.

We then assessed whether defects in phagocytic capacity might be involved in the mechanism of excessive inflammation in allergic mice with long-term exposure to *H. influenzae*. Phagocytic capacities of *H. influenzae* by both neutrophil and alveolar macrophage in BALF were assessed using flow cytometry. Neutrophils were cells highly expressed CD45 and Gr-1, and macrophages were cells highly expressed CD45 and F4/80. Phagocytosing cells were those positive for *H. influenzae*-fluorescein isothiocyanate (FITC). Phagocytic capacity was evaluated by mean fluorescence intensity (MFI) of FITC. Representative scatter plots were shown in Figure 5A.

The induction of AAD (OVA group) increased the phagocytosis by both neutrophils and macrophages in BALF (Figure 5B-5E). Compared with that, long-term exposure to *H. influenzae* during AAD (OVA/Hi group) did not suppress the proportion of phagocytosing neutrophils and macrophages (Figure 5B-5C). There was also no significant difference of MFI in neutrophils between OVA/Hi group and OVA group (Figure 5D). However, MFI in macrophages was notably decreased in allergic mice with bacterial exposure compared with those without (Figure 5E). Treatment with budesonide did not normalize the defected phagocytosis by macrophage. In contrast, after treatment with budesonide, both neutrophil and macrophage had further impairment in the phagocytosis, including both proportion of phagocytosing cell and MFI (Figure 5B-5E).

These results indicated that long-term exposure to *H. influenzae* during AAD impaired the phagocytosis by macrophage. This defect could not be normalized by budesonide treatment, which might even aggravate that.

### Long-term exposure to low-dose *H. influenzae* during allergic airway disease promotes mucus production

**Figure 6:**
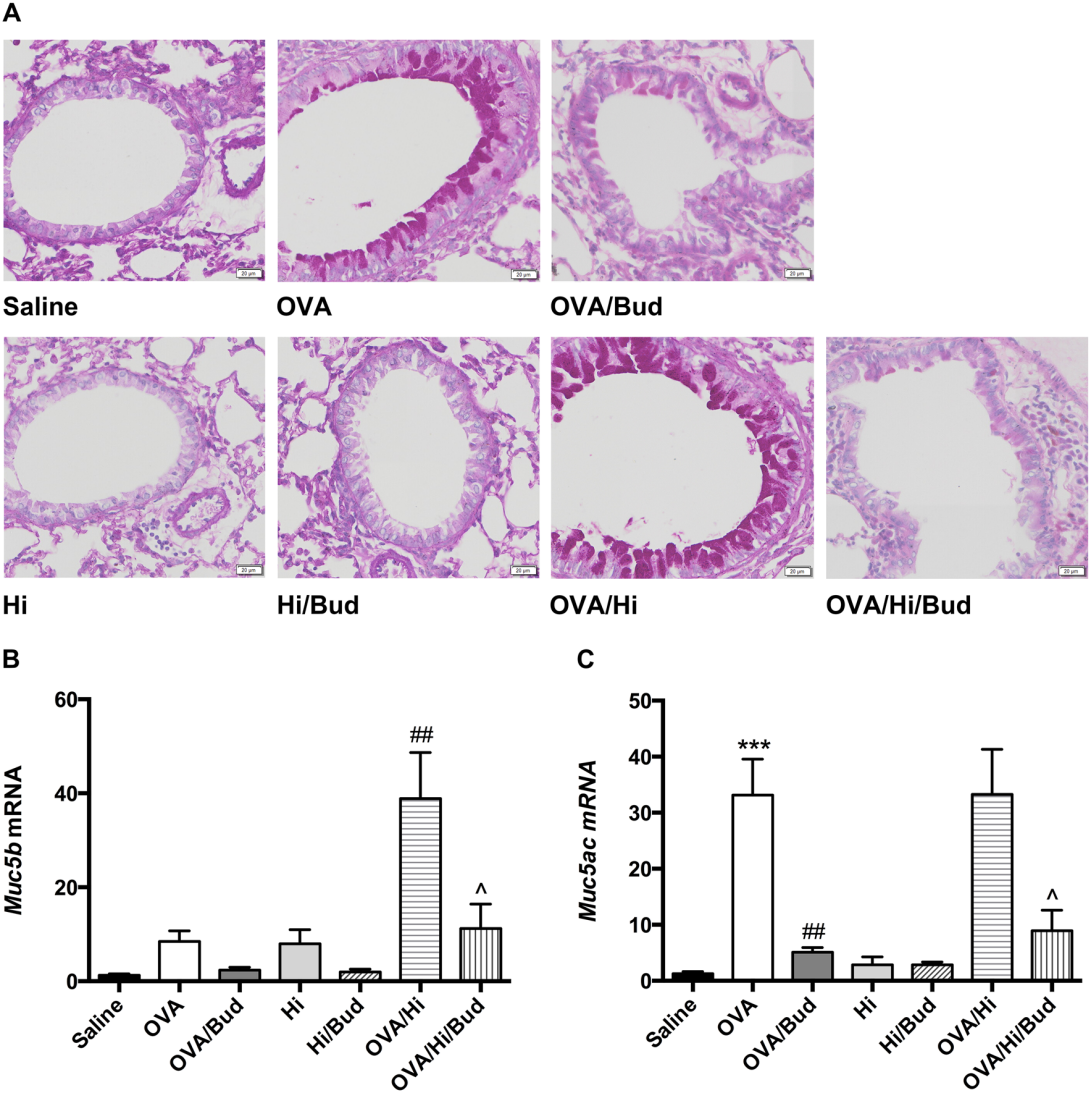
Long-term exposure to low-dose *H. influenzae* during allergic airway disease promotes mucin production Mucus production was evaluated by PAS staining (**A**, original magnification: ×200). Expressions of *Muc5b* **(B)** and *Muc5ac* **(C)** mRNAs in lung tissue were further evaluated. Data presented the mean ± SEM. Significant differences between saline group and the others are shown as ^***^P<0.001. Significant differences between OVA groups and the others are shown as ^##^P<0.01. Significant differences between OVA/Hi groups and the others are shown as ^P<0.05. Hi: *Haemophilus influenzae*; Bud: budesonide.

Mucus production helps in bacterial clearance and is involved in the host defense. Periodic acid-Schiff (PAS) staining was conducted to evaluate the mucus production in the lung tissue. Allergic mice (OVA group) exhibited a significant increase in mucus hypersecretion within the bronchi of the lungs, which resembled features of patients with asthma (Figure 6A). When combined long-term bacterial exposure with AAD, mice (OVA/Hi group) showed a further increase in mucus production with higher proportion of PAS positive cells within the bronchi than mice with AAD alone (Figure 6A).

*Muc5b* and *Muc5ac* are genes encoded structurally related mucin glycoproteins, which are the principal macromolecules in airway mucus. Therefore, we subsequently investigated their mRNA expressions in lung tissue. Notably, mice in OVA/Hi group had much higher expression of *Muc5b* but similar expression of *Muc5ac* compared with those in OVA group (Figure 6B-6C).

After treatment with budesonide, proportion of PAS positive cells in all allergic mice significantly decreased to the control level, accompanied by decreases of *Muc5b* mRNA level in OVA/Hi/Bud group and *Muc5ac* mRNA levels in both OVA/Bud and OVA/Hi/Bud groups (Figure 6A-6C).

### Long-term exposure to low-dose *H. influenzae* during allergic airway disease promotes airway remodeling

**Figure 7:**
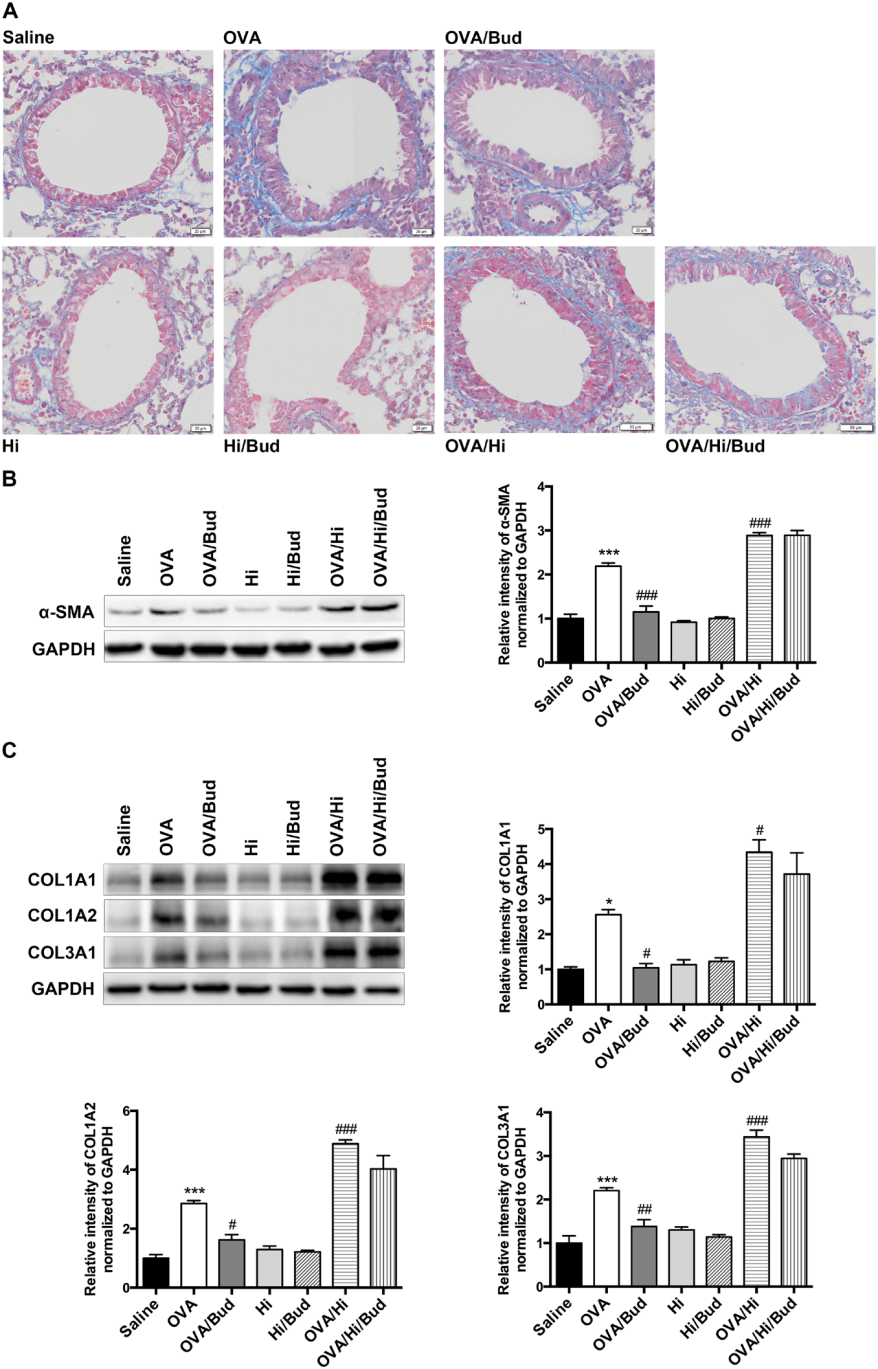
Long-term exposure to low-dose *H. influenzae* during allergic airway disease promotes airway remodeling Airway remodeling was assessed by image analysis and protein levels of markers in lung tissue. Masson staining (**A**, original magnification: OVA/Hi and OVA/Hi/Bud, ×132; others, ×200) showed collagen (blue) and smooth muscle layer (red) in the subepithelial and perivascular spaces. Western blot showed protein levels of α-SMA **(B)**, COL1A1 **(C)**, COL1A2 (C) and COL3A1 (C). Data presented the mean ± SEM. Significant differences between saline group and the others are shown as ^***^P<0.001, ^*^P<0.05. Significant differences between OVA groups and the others are shown as ^###^P<0.001, ^##^P<0.01, ^#^ P<0.05. Hi: *Haemophilus influenzae*; Bud: budesonide.

Chronic inflammation is considered as an important factor of airway remodeling, of which, collagen increase and smooth muscle hyperplasia are prominent features [22]. We sought to examine the markers of them. Collagen deposition and muscular layer thickening in subepithelial and perivascular spaces showed increases in allergic mice vs. saline controls, while they were further aggravated in allergic bacteria-exposed mice. Exposed to bacteria alone had no these effects (Figure 7A). The protein levels of markers in lung tissue revealed similar results. The increases of α-smooth muscle actin (α-SMA) and several primary types of collagens in airway remodeling in mice with AAD alone, including collagen α1 type 1 (COL1A1), collagen α2 type 1 (COL1A2) and collagen α1 type 3 (COL3A1), were robustly aggravated after long-term exposure to *H. influenzae* (OVA/Hi group vs. OVA group). Exposure to bacteria alone had no above effects (Figure 7B-7C).

Budesonide treatment showed no obvious viable effects on collagen deposition and smooth muscle hyperplasia on lung sections with Masson staining (Figure 7A). However, the protein levels of α-SMA, COL1A1, COL1A2, and COL3A1 all significantly decreased in mice with AAD alone after budesonide treatment (OVA/Bud group vs. OVA group) (Figure 7B-7C). In allergic bacteria-exposed mice, the levels of above proteins also tended to decrease but not significant (OVA/Hi/Bud group vs. OVA/Hi group) (Figure 7B-7C).

Collectively, these results indicated that long-term bacterial exposure during AAD might promote deposition of extracellular matrix and hyperplasia of smooth muscle, thus promoted airway remodeling. Budesonide treatment might remit this pro-remodeling effect, but allergic mice with long-term bacterial exposure also showed poor response to that.

## DISCUSSION

In the present study, we established a murine model of long-term airway bacterial exposure, to simulate the slight inflammation of bacterial colonization, showing that during OVA-induced AAD, long-term exposure to low-dose *H. influenzae* induced a Th17-related and steroid-resistant airway neutrophilic inflammation, and resulted in airway hypersecretion and remodeling acceleration. We also indicated that defects in Treg-associated anti-inflammatory mechanism and phagocytosis of airway macrophages might be responsible for the excessive neutrophilic inflammation.

For the establishment of the long-term model, symptoms, body weight, airway inflammation and detection of bacteria within a week after transient exposure were firstly investigated. Mice exposed to 1 × 10^6^ CFU of *H. influenzae* had no obvious symptoms and body weight changes, but most of them had persistent detectable bacterial recovery in the BALF. The accompanied airway inflammation was slight, went through an acute phase and was resolved at day 7 postinfection. We further applied this dose of *H. influenzae* to a long-term model in both non-allergic and allergic mice by repeating inoculation once a week. Similar to the outcomes induced by transient exposure, no sigh of disease, body weight loss or serious infection appeared in inoculated mice. In contrast, *H. influenzae* with the dose of 1 × 10^7^ CFU, which applied as the positive control, induced serious infections. These findings suggest that 1 × 10^6^ CFU is appropriate for the long-term model and can simulate the slight airway inflammation of colonization.

In the long-term model, we showed that in allergic mice, exposure to *H. influenzae* significantly converted the inflammatory phenotype from a Th2-associated eosinophilic inflammation to a Th17-associated neutrophilic inflammation. Notably, the intense increase of airway neutrophilic inflammation was not observed in non-allergic mice that exposed to the same dose of *H. influenzae*. This indicates that this bacteria-induced neutrophilic inflammation in the present study does not result from the infectious process and allergic airway inflammation may increase the susceptibility to that. The shift of asthma inflammatory phenotype may be attributed to the component of *H. influenzae*, lipopolysaccharide (LPS), as previously reported [23]. Although Th2 associated eosinophilic inflammation is the hallmark of allergic asthma, Th17/IL-17 is reported elevated in severe asthma [24], resulting in increased neutrophils in airways [25]. In addition, IL-17 is also reported to mediate innate and adaptive immunity to aid in host defense against bacterial infections [26]. Here, our results suggest that Th17/IL-17 might be the link between bacterial exposure and severe neutrophilic asthma.

Inhaled corticosteroids (ICS) are mainstay of asthma therapy. However, patients with neutrophilic asthma usually do not respond well to them [5]. Our results demonstrated that allergic mice with long-term exposure to *H. influenzae* showed a resistance to budesonide treatment, which resembled patients with neutrophilic asthma. This resistance may partly be attributed to Th17 cells. In patients with asthma, elevated expression of Th17 cytokine, IL-17, is found associated with neutrophilic inflammation, disease severity and high dose of steroid treatment [25]. An animal experiment shows that transfer of Th17 cells to allergic mice results in airway neutrophilic inflammation and airway hyperresponsiveness, and both of them cannot be attenuated by dexamethasone [27]. A possible mechanism for IL-17 associated glucocorticoid insensitivity is that, IL-17 is able to increase the expression of glucocorticoid receptor (GR)-β, which is an inhibitor of GR-α and can inhibit the activation of the receptor [28]. Whether long-term bacterial exposure inhibits the activation of the receptor via IL-17 induced GR-β increase needs further investigation.

Treg cells play a key role in immune tolerance and inhibit excessive inflammatory response [29]. However, compared with those in allergic mice alone, Treg-associated transcriptional factor and cytokine, *Foxp3* and IL-10, significantly down regulated in allergic mice combined with bacterial exposure. The block of IL-10 related signal may significantly augment airway neutrophilia in asthma [30]. Thus defects in Treg related anti-inflammatory mechanisms might be involved in the production of excessive neutrophilic inflammation and shift of inflammatory phenotype in mice combined with bacterial exposure and AAD. In addition, although in allergic mice, budesonide treatment significantly elevated the expression of *Foxp3* mRNA in the lung, no treatment response was found in allergic bacteria-exposed mice. Corticosteroid therapy is reported to exert its anti-inflammatory effect partly by elevating Treg cells [31]. Thus, the failure to induce Treg-associated suppression of inflammation may be also involved in steroid-resistance.

Recent data suggest that Th1-associated inflammation, which helps to combat and eliminate pathogens, also plays a role in severe steroid-resistant asthma [32]. However, in the current model, although Th1-associated factors, *Tbx21* mRNA and IFN-γ, tended to increased expressions in mice in OVA/Hi group vs. OVA group, no significances were found. Similar results were reported previously [23]. Bacterial infections may promote pathogenesis of severe asthma. However, the inflammatory response involved in the mechanism varies with the species and dose of bacteria [17, 18]. This suggests that, in addition to the common pathway, there probably exists other mechanisms depending on the species and dose of bacteria, which may contribute to the heterogenity of the inflammation. A large dose of bacteria is probably needed to promote predominant Th1 responses [33]. However, the dose of *H. influenzae* used in our model was low, insufficient to induce an active airway infection, and in this case, Th1-associated inflammation was not found significantly promoted.

Innate immunity is activated in patients with neutrophilic asthma [34]. Neutrophils and macrophages are key cellular components of innate immunity, which act in pathogens phagocytosis and elimination. Therefore, we investigated the effect of bacterial exposure in AAD on their phagocytosis. Our results demonstrated that, after long-term exposure to bacteria, the MFI of airway macrophage in allergic mice significantly decreased, indicating that although there was no change in the number of macrophage that participating in the phagocytosis, the average amount of engulfed bacteria per cell had been dramatically decreased. Similar to our results, macrophage phagocytosis is also reported impaired in severe asthma [35], non-eosinophilic asthma [36], and poorly controlled asthma [37]. The mechanism of the impaired phagocytosis in allergic bacteria-exposed mice has not been fully elucidated. It may be the direct result of bacterial exposure. But on the other hand, the innate immunity responding to LPS is impaired in asthmatic patients compared with healthy controls [38]. LPS stimulation does not alter phagocytosis of bacteria by alveolar macrophage in healthy controls but decreases the phagocytosis in moderate to severe asthmatic patients [37]. These evidences suggest that allergy itself may also play an important role in it. Therefore, the impaired phagocytosis may be due to a complex interplay between immune changes in AAD and long-term bacterial exposure. Defects in phagocytosis may lead a failure in bacterial clearance and induce the persistence of excessive inflammation. ICS treatment can not normalize the phagocytosis [39], and even further decrease it [40], consistent with what we found in the present study.

Production of mucus is one character of asthma, but also helps in bacterial clearance. We found that long-term bacterial exposure significantly increased the mucus secretion in mice with AAD. Interestingly, in allergic mice, *Muc5ac* expression was predominant, while in bacteria-exposed allergic mice, it maintained at the same level, but *Muc5b* expression showed a significant increase. This may be a response to bacteria, since it is mucin MUC5B, but not mucin MUC5AC, that contributes to mucociliary clearance [41]. Treatment with budesonide significantly inhibited the airway hypersecretion. However, this effect may also impair the mucociliary clearance. The inhibitory effects of ICS on mucociliary clearance and phagocytosis may be responsible for the increased risk of pneumonia in patients taking ICS treatments [42].

Chronic inflammation may promote airway remodeling, which will lead to irreversible airway obstruction and promote fixed airflow limitation in asthma. Extracellular matrix deposition and hyperplasia of airway smooth muscle cells are two important features of airway remodeling [22]. In allergic mice, eosinophils secrete several proteins and cytokines, such as eosinophil cationic proteins, peroxidase and transforming growth factor-β, to mediate the tissue damage and remodeling [43, 44]. When eosinophilic inflammation was inhibited by treatment with budesonide, we found a significant decrease of the synthesis of α-SMA and collagens in lung tissue. However, in bacteria-exposed allergic mice, although eosinophilic inflammation was inhibited by bacterial exposure, expression of α-SMA and collagens in the lungs significantly increased than those in allergic mice, indicating that long-term bacterial exposure notably promoted airway remodeling in AAD. On the one hand, the inhibitory effect of bacteria on eosinophilic inflammation is weaker than that of budesonide; on the other hand, bacterial exposure induces an excessive neutrophilic inflammation. Neutrophils also release key factors mediating airway remodeling, such as matrix metalloproteinase, elastase and myeloperoxidase [45]. When stimulated by LPS, neutrophils also release exosome, which contains several proteins associated with airway remodeling, and enhance hyperplasia of airway smooth muscle by exosome internalization [46]. In allergic mice combined with bacterial exposure, treatment with budesonide did not decrease the expression of α-SMA and collagens in the lung. This may be due to the steroid-resistance of the airway inflammation, and suggests that asthmatic patients with long-term bacterial colonization might not slow down the rate of progress to airway remodeling, even if taking the ICS treatments.

In conclusion, we successfully simulate the slight airway inflammation of long-term bacterial colonization in a mouse model, indicating that, during AAD, long-term exposure to a low-dose *H. influenzae* drives an increased neutrophilic airway inflammation. This inflammation is associated with an activation of Th17 immune response and a suppression of Th2 immune response, and characterized by a resistance to ICS treatment. Persistent neutrophilic inflammation may be partly attributed to defects in Treg-associated anti-inflammatory mechanism and macrophage phagocytosis, and may promote airway hypersecretion and remodeling. These characteristics resemble features of patients with neutrophilic asthma. These results contribute to our understanding of the roles of bacterial colonization in the pathogenesis and progression of neutrophilic asthma, and suggest that in asthmatic patients, airway bacterial colonization may be a potential therapeutic target. Minimizing the pathogen burden in airway, such as *H. influenzae*, may be beneficial.

## MATERIALS AND METHODS

### Mice

Female 6-8 week-old BALB/c mice were purchased from Chongqing Experimental Animal Center (Chongqing, China). Mice were maintained under pathogen-free conditions at the animal center of Xinqiao Hospital, with *ad libitum* access to food and water, and subjected to a light-dark cycle of 12 hours. The Ethics Committee of Xinqiao Hospital approved all the experiments. The use of animals in these experiments was in accordance with the guidelines issued by the Chinese Council on Animal Care and the U. S. Public Health Service Policy on Humane Care and Use of Laboratory Animals.

### Haemophilus influenzae

*Haemophilus influenzae* (ATCC 49247, USA) was grown on chocolate agar (Pangtong Co., Chongqing, China) in 5% CO_2_ at 37 °C for 20 hours. For bacterial administration, live *H. influenzae* was harvested from the plate and resuspended in saline for intranasally inoculation. For *in vitro* phagocytosis assay, harvested *H. influenzae* was heat-inactivated at 70 °C for one hour, subsequently labeled with FITC (Sigma, USA) according to manufacturer's instructions, and finally adjusted to 1 × 10^9^ CFU/ml.

### Experimental protocols

To establish an AAD, on days -19 and -12, BALB/c mice were sensitized via intraperitoneal (i.p.) injection with 100 μg of OVA (Grade V, Sigma-Aldrich, USA), absorbed to 4 mg of Imject Alum (Thermo, USA) in 200 μl of a sterilized 0.9% saline. Beginning on day -5, the mice were challenged through the respiratory tract with 1% aerosolized OVA in saline for 30 minutes three times a week. Non-allergic controls were received saline sensitization and challenge.

Twenty-four hours after the third challenge every week, mice were anesthetized via i.p. injection with 50 mg/kg of pentobarbital (Dingguo Co., Ltd., Beijing, China) and intranasally inoculated with live *H. influenzae*. Appropriate dose for the model of long-term exposure would be determined through a transient exposure in non-allergic mice. Saline control group and AAD alone group were intranasally instilled with saline instead.

Budesonide treatment was performed as previously reported with minor modification [47]. Briefly, mice were treated nasally with nebulized budesonide once per day for 6 consecutive days in the last week just before endpoint, with the manner similar to that of OVA challenge. Budesonide was diluted to 0.125 mg/ml. Each mouse was allowed to breath in the aerosol for 2 minutes. The dose given to each mouse was thus equivalent to a 1000 μg dose for an adult. Untreated mouse were exposed to saline.

Seven days after the 8th colonization and 24 hours after the last challenge (day 56), mice were sacrificed via lethally anesthetization for analyses.

### Body weight

Body weights were measured before every inoculation and just before the final sacrifice.

### Bronchoalveolar lavage

Bronchoalveolar lavage was performed as described before [23]. Briefly, a tube was inserted into the trachea and lavaging was performed three times consecutively with 1 ml of ice-cold saline. A total of 0.15 ml of BALF was removed for bacterial recovery. Total cell counting was then performed using a haemocytometer. After that, the remaining BALF was centrifuged at 400 × g at 4 °C for 5 minutes. Cell pellets were resuspended with phosphate-buffered saline (PBS, HyClone, USA) to a concentration of 1 × 10^7^/ml for subsequent flow cytometry analysis and cell differential counting via Wright-Giemsa staining (BASO Co., Zhuhai, China). Supernatants were harvested and stored at -80 °C until cytokines analyses.

### Bacterial recovery

A total of 0.1 ml of serial dilutions of BALF were plated onto chocolate agar plates (Pangtong Co., Chongqing, China) and incubated in 5% CO_2_ at 37 °C for 24-48 hours. Colonies were enumerated and then the actual bacterial CFU were calculated in BALF.

### Flow cytometry

A flow cytometric method was used to determine the phagocytic capacity of *H. influenzae* by neutrophils and alveolar macrophages. FITC labeled *H. influenzae* was adjusted to 1 × 10^9^ CFU/ml and added to resuspended BALF cells (1 × 10^7^/ml). After incubated at 37 °C for 10 minutes, cells were washed with PBS to remove potentially excessive extracellular bacteria and subsequently stained with the following fluorochrome-labeled antibodies (all obtained from Biolegend, USA): PerCP/Cy5.5-CD45, PE-F4/80 and APC-Cy7-Gr1. The cells were incubated with these antibodies for 30 minutes at 4 °C in the dark. Then they were washed, resuspended with PBS and detected using a Beckman Coulter (Galios, USA) flow cytometry. Among all the cells positive for CD45, neutrophils were defined by positive staining for Gr1 and alveolar macrophage were defined by positive staining for F4/80. Phagocytosing cells were those positive for *H. influenzae*-FITC. Phagocytic capacity was evaluated by MFI of FITC. Flow cytometry data were analysed with Flowjo software (version 10.0.7).

### Cytokine analysis

Cytokine levels were determined using a Mouse High Sensitivity T Cell Magnetic Bead Panel kit (Merck Millipore, Germany) according to the manufacturer's instructions.

### Lung histology

The left lungs were harvested, fixed with 4% paraformaldehyde and embedded in paraffin after dehydration. Then, 6 μm sections of embedded lung tissue were mounted onto slides and stained with hematoxylin and eosin to identify tissue inflammation, with PAS reagent to identify mucus production, and with Masson trichrome to evaluate the collagen deposition and smooth muscle hyperplasia. Tissue sections were viewed with Olympus BX61VS microscope (Japan) and OlyVIA software (Japan).

### Reverse transcription-polymerase chain reaction (RT-PCR)

Lower lobe of right lungs were removed and immersed in RNA*later*™ Solution (Invitrogen, USA). Total RNA was extracted using TRIzol™ Reagent (Invitrogen, USA). cDNA was prepared from total RNA using QuantiNova™ Reverse Transcription Kit (Qiagen, Germany) according to the manufacturer's instructions. Quantitative PCR was performed using QuantiNova™ SYBR^®^ Green PCR kit (Qiagen, Germany) on Rotor Gene (Qiagen, Germany) according to the manufacturer's instructions. Primers (all synthesized by SANGON Biotech, Shanghai, China) were: mouse *Gapdh*, F 5’-GACGGCCGCATCTTCTTGT-3’, R 5’-ACACCGACCTTCACCATTTTGT-3’; mouse *Tbx21*, F 5’-AGCAAGGACGGCGAATGTT-3’, R 5’-GGGTGGACATATAAGCGGTTC-3’; mouse *Gata3*, F 5’-CTCGGCCATTCGTACATGGAA-3’, R 5’-GGATACCTCTGCACCGTAGC-3’; mouse *Rorγt*, F 5’-AGCACTGACGGCCAACTTACTC-3’, R 5’-CGCTGCCGTAGAAGGTCCTC-3’; mouse *Foxp3*, F 5’-CCCTTTCACCTATGCCACCCTTATC-3’, R 5’-GGCGGGGTGGTTTCTGAAGTAGG-3’; mouse *Muc5b*, F 5’-TCACCGGAGACAGTCAGAGAG-3’, R 5’-GGTGTAAGGCGCTCATGCTA-3’; mouse *Muc5ac*, F 5’-CACTGGAGCTGGATGTCAGA-3’, R 5’-ACACAGCCTCCATTTCCATC-3’.

### Western blot

Upper lobe of right lungs were removed and homogenized in a tissue protein extraction reagent (Thermo, USA). The lysates were centrifuged at 16000 × g at 4 °C for 15 minutes to remove insoluble protein. The protein concentrations were determined and subsequently separated using SDS-PAGE. Separated proteins were transferred to PVDF membranes (Millipore, Germany), blocked with 5% skimmed milk, and then incubated at 4 °C overnight using the following primary antibodies: α-SMA (ab5649, Abcam, USA), COL1A1 (sc-293182, Santa Cruz, USA), COL1A2 (sc-393537, Santa Cruz, USA), COL3A1 (sc-271249, Santa Cruz, USA), GAPDH (60004, Proteintech, USA). The membranes were washed three times, incubated for 1 hour using horseradish peroxidase-conjugated secondary antibodies and then visualized using an enhanced chemiluminescence detection kit (Amersham Pharmacia, Piscataway, NJ).

### Statistical analysis

Data were presented as the mean ± standard error of the mean (SEM). Statistical analyses for multiple comparisons were performed via one-way ANOVA with the Bonferronni post-test using Graphpad Prism (version 6). One-way repeated measures ANOVA was used to analyze body weight data using IBM SPSS Statistics software (version 22.0). P < 0.05 were considered statistically significant.

## Abbreviations

AAD: allergic airway disease
OVA: ovalbumin
Th: T helper
Treg: regulatory T cell
BALF: bronchoalveolar lavage fluids
CFU: colony-forming units
IL: interleukin
KC: keratinocyte chemokine
IFN: interferon
FITC: fluorescein isothiocyanate
MFI: mean fluorescence intensity
PAS: Periodic acid-Schiff
α-SMA: α-smooth muscle actin
COL1A1: collagen α1 type 1
COL1A2: collagen α2 type 1
COL3A1: collagen α1 type 3
LPS: lipopolysaccharide
ICS: inhaled corticosteroid
GR: glucocorticoid receptor
PBS: phosphate-buffered saline
RT-PCR: reverse transcription-polymerase chain reaction
SEM: standard error of the mean
